# New tricks for KDEL receptors

**DOI:** 10.18632/oncotarget.5444

**Published:** 2015-08-31

**Authors:** Ming Yuan Li, Roberto Bruzzone, Pei Gang Wang

**Affiliations:** HKU-Pasteur Research Pole, School of Public Health, LKS Faculty of Medicine, The University of Hong Kong, Hong Kong SAR; Key Laboratory of Protein and Peptide Pharmaceuticals, Institute of Biophysics, Chinese Academy of Sciences, Beijing, PR China

**Keywords:** KDEL receptors, dengue virus, receptor, egress, extracellular matrix degradation

The function of KDEL receptors (KDELR) is to capture endoplasmic reticulum (ER)-resident chaperones in the acidic environment of the Golgi apparatus, by recognizing their C-terminal motif, and retrieve them back to the ER [1]. Certain structural features of KDELR, for example a similar topology to that of G-protein-coupled receptors (GPCR), a large family of proteins involved in virtually all pathways of cellular signaling, hinted to a more sophisticated activity. Specifically, binding of chaperones to KDELR recruits additional G proteins and trigger signaling pathways that result in the activation of both retrograde and anterograde transport, effectively dispatching the incoming membraane load at the Golgi during activated secretion [2, 3]. These findings place KDLER at the center of a complex system of signals, which originate at the Golgi but exert their effects at multiple levels. Two recent papers have shed new light on the role of KDELR by implicating them in as diverse functions as cell invasion [4] and virus infection [5].

The traffic sensor hypothesis posits that, in the Golgi, chaperones bind to KDELR and trigger a G_q_-dependent activation of Src, accelerating anterograde transport [2]. Ruggiero et al. zoomed in on the role of this molecular device in invadopodia formation, a Src-dependent process that requires activation of the exocytic machinery for extracellular matrix (ECM) degradation and invasive growth [4]. They found that persistent stimulation of KDELR increased ECM degradation, and that inhibition produced the expected opposite effect. These observations correlated with the presence of phosphorylated Src in the regions overlapping ECM degradation patches. KDLER stimulation led to tyrosine phosphorylation of ASAP1, a known Src effector, and experimental manipulations leading to either activation or inhibition of ASAP1 resulted in the predicted effect on ECM degradation. If Src regulates the signaling pathway leading to ECM degradation, however, any change in its activity will impact this process. The authors speculate that a KDELR-dependent mechanism may be operational under specific circumstances, because the microenvironment of solid tumors is characterized by low oxygen, which, in turn, triggers the unfolded protein response and chaperone overexpression. This would be equivalent to a chronic stimulation of KDELR by an overabundance of ligands, activating a signaling circuit that links KDELR to Src phosphorylation, invadopodia formation and ECM degradation, facilitating tumor dissemination. This possibility will no doubt be tested in more relevant cellular and animal models.

KDELR travel without cargo to the Golgi because binding to the KDEL motif is weak at the neutral pH of the ER. This is an ideal situation to be exploited by professional parasites, such as viruses, which led us to consider the involvement of KDELR in assisting Dengue virus (DENV) secretion [5]. DENV is responsible for 50-100 million infections annually in over 100 endemic countries [6]. Viral particles assemble and bud into the ER lumen, translocate to the Golgi to complete their maturation and are released by exocytosis. During infection there is a massive overproduction of cargo (newly assembled virions in the ER) that needs to be coupled to accelerated transportation. We had previously produced DENV recombinant subviral particles (RSPs) by expressing the two major structural glycoproteins, Envelope (E) and pre-Membrane (prM), and showed that RSPs can be used as proxies to mimic the secretion of DENV. Using RSPs, we demonstrated that sequestering KDELR in Golgi apparatus by depletion of the small GTPases Arf4 and Arf5 accumulated RSPs in the ER [7]. We have now found that KDELR knockdown by siRNA reduced egress of RSPs and DENV and have identified three positive charged amino acid residues at the N-terminus of prM that mediate interaction with KDELR in vitro [5]. Only KDELR1 and 2 were involved, similarly to what observed for ECM degradation [4]. However, an unexpected level of specificity was observed in that DENV4, one of the four serotypes, and West Nile virus, a member of the same *Flaviviridae* family, was not affected by KDELR depletion, suggesting that there might be some alternatives to KDELR-assisted transportation, an area that we are actively investigating.

These findings identify KDELR as the first intracellular receptor required for viral egress. Although viral secretion and entry are two different processes with opposite direction, the role of KDELR as a luminal receptor shares several similarities with cell surface proteins that capture viral particles to mediate their internalization via endocytosis. In both cases they carry viruses from a neutral environment (extracellular milieu or ER) to an acidic compartment (endosome or Golgi), help them to overcome a lipid membrane barrier to reach the final destination (cytoplasm for replication or extracellular milieu for another infection round) and ensure sorting of viral cargos along the correct compartment. The mechanism that controls the dissociation of viral particles from KDELR is unknown, but analogies to dissociation of receptor-ligand complexes in endosomes may be drawn. As the three key positively charged amino acids that we mutated have a p*I* close to the pH in the Golgi, it is tempting to speculate that the acidic environment in Golgi may facilitate dissociation of DENV from KDELR by reducing their binding affinity. By contrast, the canonical KDEL motif that is recognized by KDELR in Golgi is more negatively charged and this difference may underlie the switching of cargo preference that allows retrieving of ER-resident proteins from Golgi. Because KDELR functions as a signal transduction module, it is possible that interaction between DENV and KDELR may also result in GPCR activation, as shown in the Golgi [3], to initiate cargo-dependent transport and stimulate a greater flow of vesicles that are required for efficient transportation of newly formed DENV along the secretory pathway. These observations lend further support to the role of KDELR in regulating endomembrane dynamics and provide a conceptual framework to address the mechanism underlying membrane fluxes in health and disease.
